# 7-Ethyl-10-Hydroxycamptothecin, a DNA Topoisomerase I Inhibitor, Performs BRD4 Inhibitory Activity and Inhibits Human Leukemic Cell Growth

**DOI:** 10.3389/fphar.2021.664176

**Published:** 2021-04-29

**Authors:** Airong Wang, Lingling Li, Mengya Li, Shujuan Wang, Chong Wang

**Affiliations:** Department of Hematology, The First Affiliated Hospital of Zhengzhou University, Zhengzhou, China

**Keywords:** SN-38, BRD4, inhibitor, leukemic cell, growth

## Abstract

7-Ethyl-10-hydroxycamptothecin (SN-38) is an active metabolite of CPT-11, which can inhibit DNA topoisomerase I, DNA synthesis and cause frequent DNA single-strand breaks. In our study, SN-38 was characterized as a potent and reversible BRD4 inhibitor [IC_50_ = 660.2 nM against BRD4 (BD1) and IC_50_ = 547.7 nM against BRD4 (BD2)] in biochemical assay using drug repurposing strategy. Additional cellular assay suggested that SN-38 can bind BRD4 in human leukemic cell K562 and inhibit cell growth with IC_50_ = 0.2798 μM in a BRD4 dependent manner partially. Additionally, mechanism study indicated that SN-38 can induce the accumulation of BRD4 substrate c-Myc and cleavage of caspase 3. In sum, our findings identified BRD4 as a new target of SN-38 and reveals SN-38 as a modifier of histone acetylation reader for the first time, which may provide a new insight for further optimization of dual target inhibitor.

## Introduction

Epigenetics is the study of heritable phenotype changes that do not involve alterations in the DNA sequence. While genetic changes can alter which protein is produced, epigenetic changes affect gene expression to turn genes “on” and “off.” Among the diverse kinds of epigenetics, histone modification is the key player in regulating gene expression, including methylation, phosphorylation, acetylation, ubiquitylation, and sumoylation (Feinberg and Tycko, 2004). These covalent post-translational modification (PTM) on histone proteins can impact gene expression by altering chromatin structure or recruiting histone modifiers.

There are three groups of epigenetic modifiers, and referred to as “writers,” “erasers,” and “readers” (Marmorstein and Roth, 2001). As a family of key proteins in epigenetics, bromodomain (BRD)-containing proteins family can specifically recognize and bind acetylated lysine on histones as a readers of lysine acetylation (Filippakopoulos and Knapp, 2014). The BRD family is highly conserved during evolution, and the bromodomain and terminal outer domain (BET) protein family belongs to the BRD protein family. It is composed of bromodomain-containing protein 2 (BRD2), bromodomain-containing protein 3 (BRD3), bromodomain-containing protein 4 (BRD4) and bromodomain testis-specific protein (BRDT) consists of four members, each of which contains two tandem BRDs (BD1 and BD2) and an extra terminal domain (ET) (Wu and Chiang, 2007; Filippakopoulos et al., 2012; Taniguchi, 2016). BRD4 is currently the most widely studied member of the BRD family. It can recognize and bind histone and non-histone acetylated lysine to participate in the development of many diseases (Moriniere et al., 2009). Recent reports indicating that BRD4 is highly expressed in various cancers, including gastric cancer, breast cancer, leukemia and so on (Sahai et al., 2016; Kulikowski et al., 2021), and is involved in regulating cell growth, cell cycle and apoptosis, and other cell processes. Its imbalance is related to the occurrence and development of many cancers. In addition, BRD4 can recruit and activate p-TEFb (transcription elongation factor b), and activated p-TEFb can phosphorylate RNA Pol II (Itzen et al., 2014; Fujinaga et al., 2015), thereby promoting oncogenes (c-MYC) transcription extension (Roe et al., 2015).

Until now, a large number of studies have shown that BRD4 inhibitor has shown good antitumor activity in a variety of malignancies, and a variety of BRD4 inhibitor has been used in clinical trials of hematological malignancies, solid tumors, inflammation and cardiovascular diseases (Schaefer, 2014), and several inhibitors have entered the clinical phase (Theodoulou et al., 2016; Alqahtani et al., 2019), including OTX015 (NCT02259114), GSK525762 (NCT01587703), TEN-010 (NCT02308761) and CPI-0610 (NCT01949883 and NCT02157636), they were used to treat lymphoma, acute myeloid leukemia, multiple myeloma and myelodysplastic syndrome, etc. Unfortunately, none of them have entered clinical stages in recent years (Andrieu et al., 2016). So, new potent BRD4 inhibitor with novel skeleton is still in needed.

In this present study, SN-38 was identified by high throughput screening with BRD4 inhibitor screening assay from a small compound library. Then, SN-38 was characterized to inhibit BRD4(BD1) and BRD4(BD2) potently and reversibly. Additional cellular study suggested that SN-38 can suppress the proliferation of chronic myelogenous leukaemia (CML) cell line K562. In a nutshell, this finding gave a support that SN-38 may serve as a lead compound to inhibit CML proliferation by targeting BRD4.

## Materials and Methods

### Cell Culture and Materials

K562 cell line was obtained from National Collection of Authenticated Cell Cultures (Shanghai, China) and cultured in Roswell Park Memorial Institute (RPMI) 1640 medium (Solarbio, China) supplemented with 10% fetal bovine serum (FBS) (Biological Industries, Israel) and 1% penicillin-streptomycin. Cells were maintained at 37°C in a humidified atmosphere of 95% air and 5% CO_2_.

Raloxifene was purchased from commercial source (MB1615, meilunbio, China) and the purity is above 98% and prepared at concentration of 10 mM with dimethyl sulfoxide (DMSO) for stock. The highest DMSO concentration in the medium was <0.1% v/v, which had no any substantial effect on the cell.

### Cell Viability Assay

CCK-8 (Beyotime Biotechnology, China) was used to determine cell viability. Cells were seeded in 96-well plates at a density of 2,000 per well, and treated with series of compounds. After 48, 72 or 96 h incubation, 10 μl CCK-8 solution was added to each well and incubated for additional 3 h at 37°C. Then the plate was read by multiplate reader (PE Envision, United States) at 570 nm. IC_50_ was calculated by the GraphPad Prism 9.0 software.

### Expression and Purification of BRD4(BD1) and BRD4(BD2)

Genes encoding BRD4(BD1) and BRD4(BD2) were cloned into pGEX-4T-1 plasmid by double digestion, respectively. Then, the reconstructed plasmid was transfected into *E. coli* BL21(DE3) cells, and grown overnight at 37°C in 50 ml of Terrific Broth (TB) medium. Then the medium was diluted 100-fold in 1 L of fresh TB medium and cell growth was at 37°C to an optical density of about 0.8 at OD600 before the temperature was decreased to 15°C. Then, cells were grown overnight at 15°C in the presence of 0.5 mM isopropyl-β-D-thiogalactopyranoside (IPTG). Cells were collected by centrifugation (5,000 g for 15 min at 4°C) and resuspended in lysis buffer [50 mM tris (hydroxymethyl)aminomethane (Tris), pH 7.3, 300 mM NaCl, 10 mM imidazole, 5% glycerol with freshly added 0.5 mM tris (2-carboxyethyl) phosphine hydrochloride (TCEP), and 1 mM phenylmethanesulfonyl fluoride (PMSF)] and lyzed using sonication. The lysate was then cleared by centrifugation (12,000 g for 1 h at 4°C) and was applied to Glutathione Sepharose 4B resin for further purification. After washing with the binding buffer (50 mM Tris, pH 7.3, 300 mM NaCl), the target protein was eluted with elution buffer (50 mM Tris, pH 7.3, 300 mM NaCl, 10 mM reduced glutathione). All fractions were collected and monitored by SDS−polyacrylamide gel electrophoresis. After the addition of 1 mM dithiothreitol (DTT), the protein was used for further enzymatic assay.

### BRD4 Assay

Compounds were evaluated in biochemical bromodomain binding assays. The acetylated histone H4 (H4K5acK8acK12acK16ac) was synthesized as a substrate, Eu^3+^ cryptate and d2 beads (Cisbio, France) were used as energy donor and energy acceptor, respectively. BRD4 (BD1) or BRD4 (BD2), acetylated histone H4, candidate compound and buffer are present in the 20 μl system. After incubated 30 min in 25°C, fluorescence signal was detected by microplate reader (Envision, PerkinElmer, United States) with excitation at 320 nm, emission at 665 and 615 nm. The final TR-FRET signal was signal 665 nm/signal 615 nm × 10,000 nm. Test compounds that compete with the biotinylated ligand for BRD4 binding can reduce the TR-FRET signal. % inhibition = [(compound signal) − (min signal)]/[(max signal) − (min signal)] × 100. IC_50_ was analyzed by GraphPad 9.0 (La Jolla, CA, United States).

### Dialysis Experiment

The BRD4 (BD1) or BRD4 (BD2) recombinant was incubated with 100 folds of the IC_50_ of candidate compound at 37°C for 30 min, then the mixture was dialyzed against the assay buffer with five times changing for each 2 h. Finally, the mixture before and after dialysis was subjected to test the activity of BRD4 with above mentioned TR-FRET assay. JQ-1 was used as a positive control.

### Dilution Assay

The BRD4 (BD1) or BRD4 (BD2) recombinant was incubated with 100 folds of the IC_50_ of candidate compound at 37°C for 30 min, then the mixture was diluted 100 folds to test the activity of BRD4 with above mentioned TR-FRET assay. JQ-1 was used as a positive control.

### Protein Thermal Shift Assay

BRD4(BD1) or BRD4(BD2), SYPRO Orange (Thermo Fisher, United States), candidate compound and PBS buffer are present in the 20 μl system. Then real-time PCR instrument (QuantStudio 7, Applied Biosystems, United States) was used to heat from 25 to 95°C and detect fluorescence signal. d (Fluorescence)/dT and ΔTm was analyzed.

### Cellular Thermal Shift Assay (CETSA)

CETSA (Martinez Molina and Nordlund, 2016) was performed to confirm the cellular interaction between BRD4 and SN-38 *in vitro*. The K562 cells were collected and suspended in the PBS. Then, cell suspension was freeze-thawed repeatedly for three times using liquid nitrogen, and the supernatant was collected by centrifuge at 20,000 g for 20 min at 4°C. After that, the protein was incubated with SN-38 for 30 min, and divided into six tubes in average. The tube was then heated at indicated temperature for 3 min, and cooled for 3 min. The supernatant was then transferred to a new microtubule after centrifuge at 20,000 g for 20 min at 4°C, and analyzed by sodium dodecyl sulfate polyacrylamide gel electrophoresis (SDS-PAGE) followed by western blot analysis.

### Western Blotting Analysis

Using radio immunoprecipitation assay (RIPA) buffer to lysate cells, the protein concentration was then determined by bicinchoninic acid (BCA) method. After that, the collected protein was denatured in the presence of loading buffer (Takara, Japan) and subjected to SDS-PAGE with same amount. After running, the isolated protein on gel was transferred to the 0.2 μm nitrocellulose (NC) membrane (Pall, United States). Following the blockage with 5% milk, the membrane was incubated with primary antibody and secondary antibody, respectively. Finally, the membrane was imaged with X-ray film (Kodak, Japan) with the help of electrochemiluminescence (ECL) imaging kit (ThermoFisher, United States). The antibodies used in this study are as follows: Ki67 antibody (HuaBio, ET1609-34, China), BRD4 Antibody (AFFINITY, DF2905, United States), C-MYC antibody (HuaBio, 0912-2, China), active + pro caspase-3 antibody (HuaBio, ET1608-64, China), GAPDH antibody (Goodhere, AB-P-R001, China), Bcl-XL antibody (HuaBio, ET1603-28, China), BAX antibody (Proteintech, 50599-2-Ig, United States), peroxidase affiniPure goat anti-rabbit IgG (H + L) (Jackson Immumo Research, 111-035-003, United States).

### Apoptotic Analysis

The apoptosis was quantified by fluorescence activated cell sorting (FACS) with Annexin V-FITC/PI staining kit from BioVision (United States). Ten-thousand events for each sample were counted and analyzed by Accuri C6 flow cytometer (BD, United States). The early and late apoptotic cells were identified by the localization of Annexin V and PI.

### Molecular Docking

Crystal structure of the first bromodomain of BRD4 (PDB: 6CD4) was obtained from the RCSB Protein Data Bank (Wang et al., 2018). Molecular docking was performed by Molecular Operating Environment (MOE) modeling software 2014.0901. The process includes the preparation of the protein and ligand and docking. The preparation of BRD4 was performed as follows, deletion of water molecules, adding hydrogen atoms, assigning the partial charges and energy minimization. The ligand binding pocket residues of BRD4 were identified by Site Finder from MOE. And chemical structures of SN-38 were input as mol2 format, and the conformation was optimized using energy minimization and conformational search. Finally, SN-38 was docked into BRD4 using Dock from MOE. And favorable ligand conformation is selected and analyzed using Discovery Studio Visualizer 4.5.

### Statistical Analysis

Data was analyzed by GraphPad Prism Version 9.0. *t*-test or one-way analysis of variance (ANOVA) was used for statistical significance evaluation and **p < 0.01 was considered statistically significant.

## Results and Discussion

### SN-38 was Identified as a Novel Potent BRD4 Inhibitor Using Drug Repurposing Strategy

**FIGURE 1:**
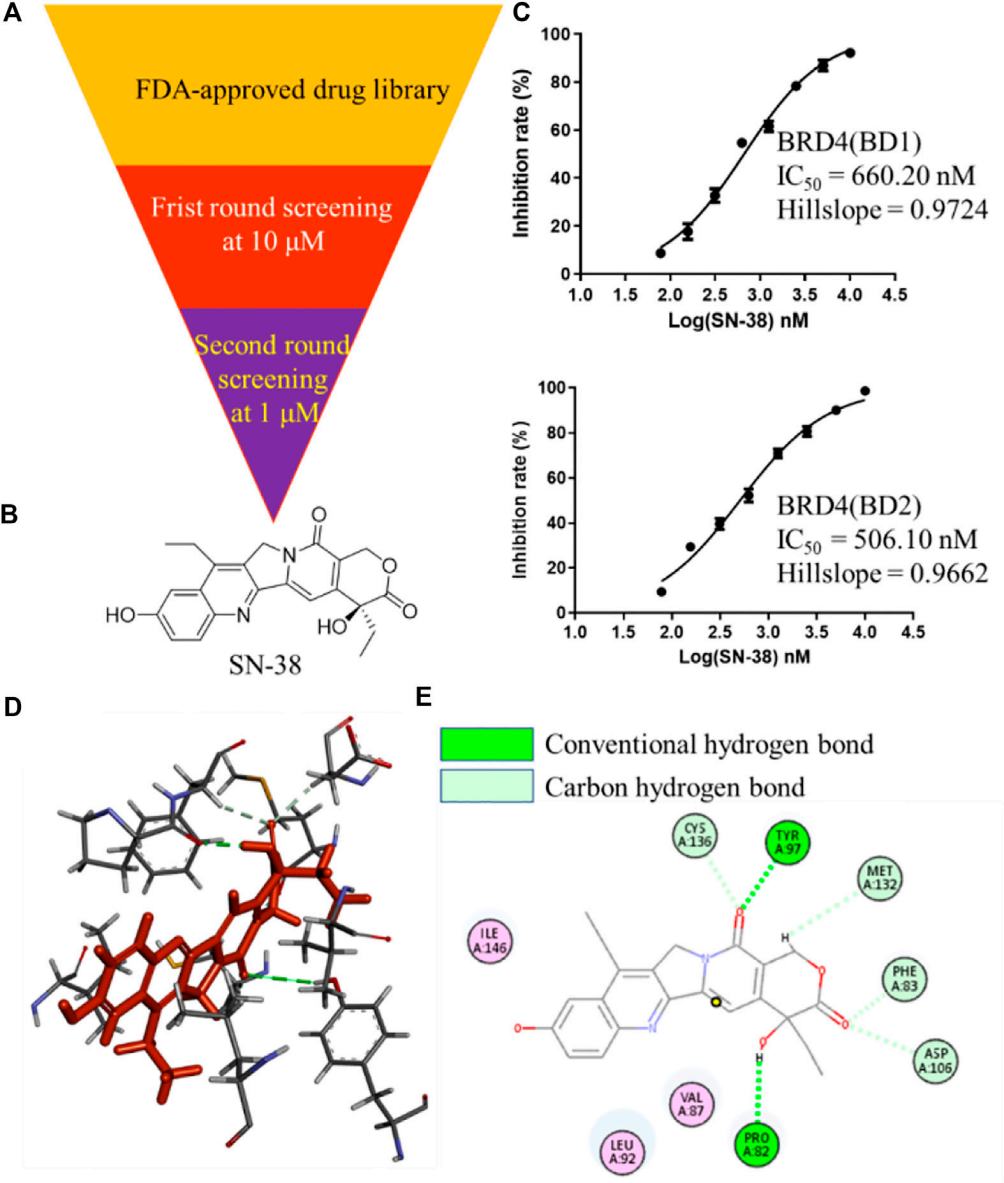
Identification of SN-38 as a potent BRD4 inhibitor. **(A)** Flowchart about the identification of SN-38 as a BRD4 inhibitor. **(B)** Structure of SN-38; **(C)** Inhibition curve of SN-38 against BRD4(BD1) **(up)** and BRD4 (BD2) **(down)** recombinant. **(D)** Molecular modeling of SN-38 in the first bromodomain of BRD4 (PDB: 6CD4); **(E)** he Hydrogen bond interaction between BRD4 and SN-38 (PDB: 6CD4). Data were shown as mean ± SD with three times replication.

To identify BRD4 inhibitor with novel skeleton, a Food and Drug Administration (FDA)-approved drug library containing 2,864 compounds from SelleckChem was applied to BRD4 screening assay using Time-Resolved Fluorescence Resonance Energy Transfer (TR-FRET) method (Figure 1A). In the first round, all candidate compounds were subjected to BRD4 (BD1) assay at 10 μM. Then, compounds that can inhibit BRD4 (BD1) activity with more than 50% were collected and subjected to the second-round screening at 1 μM. After that, compounds that can inhibit BRD4 (BD1) activity with more than 50% at 1 μM were subjected to the IC_50_ evaluation, and SN-38 (Figure 1B) was characterized as one of the most potent BRD4 (BD1) inhibitor with IC_50_ = 660.2 nM with hillslope = 0.9724 (Figure 1C), while JQ-1 can inhibit BRD4 (BD1) with IC_50_ = 163.2 nM. In addition, SN-38 can also inhibit BRD4 (BD2) with IC_50_ = 506.1 nM with hillslope = 0.9562 (Figure 1C), while JQ-1 can inhibit BRD4(BD1) with IC_50_ = 123.9 nM. To further evaluate the binding property of SN-38 to BRD4, the protein thermal shift assay (Huynh and Partch, 2015) was used to detect the ΔTm between the Tm (BRD4 + 6 μM SN-38) value and the blank Tm (BRD4 + DMSO), using JQ-1 as the control compound. As indicated in Table 1, for BRD4 (BD1) and BRD4 (BD2), the ΔTm increased upon SN-38 and JQ-1 treatment. The results suggest that JQ-1 and SN-38 can enhance the stability of BRD4 (BD1) and BRD4 (BD2) recombinant. Molecular modeling also suggested that SN-38 can interact with first bromodomain of BRD4 (Figure 1D) and form classical hydrogen bond with TYR97 and PRO82 (Figure 1E), respectively, indicating the importance of carbonyl and hydroxy group of SN-38. Meanwhile, several carbon hydrogen bonds were also formed (Figure 1E). All these findings confirmed that SN-38 can bind and inhibit BRD4 potently.

**TABLE 1:** Protein thermal shift assay performed with SN-38 and JQ-1.

	Compound	ΔTm (°C)
BRD4(BD1)	JQ-1	9.4
	SN-38	5.8
BRD4(BD2)	JQ-1	11.6
	SN-38	6.3

### SN-38 Inhibited BRD4 in a Reversible Manner and Inhibit K562 Proliferation

**FIGURE 2:**
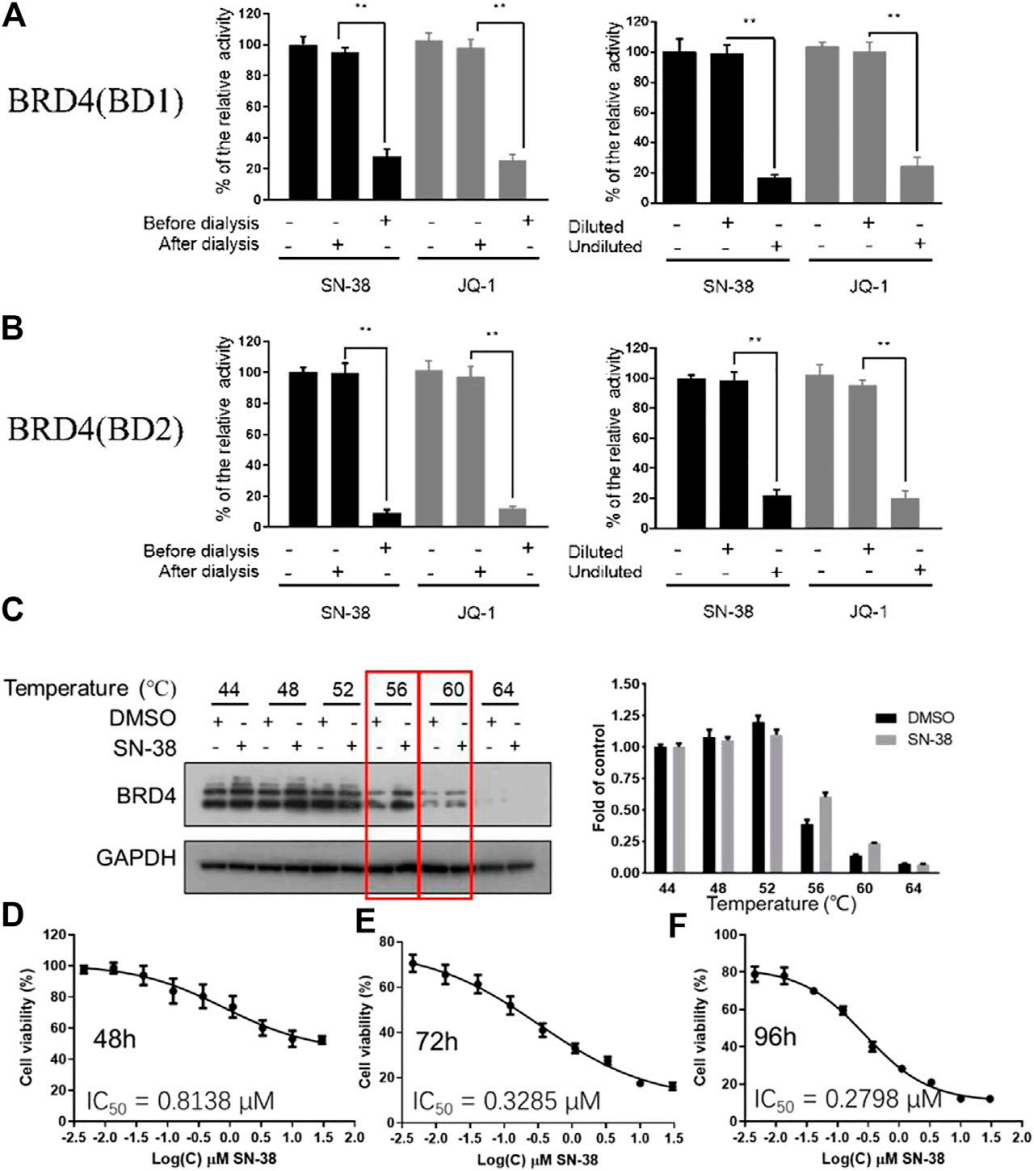
SN-38 inhibited BRD4 (BD1) and BRD4 (BD1) in a reversible manner, SN-38 can bind BRD4 and inhibit cell proliferation in K562 cells. **(A,B)** Reversible test of SN-38 against BRD4 (BD1) **(up)** and BRD4 (BD2) **(down)** recombinant using dialysis assay and dilution assay. JQ-1 was used as a positive control. **(C)** Protein levels of BRD4 in K562 cells treated with SN-38 using CETSA. **(D–F)** Cell viability of K562 cells that exposed to SN-38 fir 48 h **(D)**, 72 h **(E)** or 96 h **(F)**, respectively. **p < 0.01 were considered statistically significant compared diluted group vs. undiluted group. Data were shown as mean ± SD with three times replication.

As we have confirmed that SN-38 can inhibit BRD4 potently, whether this process is reversible or not remains unknown. So, to test the reversibility of SN-38 against the binding between BRD4 and its histone substrate, dialysis experiment and dilution assay were applied. After the co-incubation of BRD4 (BD1) recombinant and 33 μM SN-38, the mixture was subjected to BRD4 (BD1) assay, and result in Figures 2A,B suggested that 33 μM SN-38 can inhibit BRD4 (BD1) completely, while depletion of SN-38 by dialysis can recover the activity of BRD4 (BD1), indicating that SN-38 may be a reversible inhibitor against BRD4 (BD1). To further support this result, dilution assay was also applied. While the recombinant BRD4 (BD1) was incubated with SN-38 at the concentration of 33 μM for 30 min, then the mixture was diluted 100-fold and subjected to the BRD4 (BD1) assay. As shown in Figures 2A,B, after the dilution, the enzymatic activity was significantly restored, which further suggested that SN-38 is a reversible BRD4 (BD1) inhibitor. Additional dialysis experiment and dilution assay using BRD4 (BD2) assay also gave solid evidence that SN-38 is not only a reversible BRD4 (BD1) inhibitor, but also inhibited BRD4 (BD2) reversibly.

As we have confirmed that SN-38 can bind and inhibit BRD4 potently in a reversible manner, whether SN-38 can interact with BRD4 in cellular level is still unknown. So, to answer this question, CETSA was performed in K562 cells in the presence of SN-38 at different temperature. As indicated in Figure 2C, when the protein in K562 was denatured at 56 and 60°C in the presence of SN-38, BRD4 protein can be stabilized, indicating the cellular target engagement of SN-38 in K562 cells. Further cell viability suggested that SN-38 can inhibit K562 cell proliferation with IC_50_ = 0.8138 μM for 48 h treatment (Figure 2D), IC_50_ = 0.3285 μM for 72 h treatment (Figure 2E), IC_50_ = 0.2798 μM for 96 h treatment (Figure 2F). All data above indicated that SN-38 can bind BRD4 and inhibit K562 proliferation.

### SN-38 can Induce the Apoptosis of K562 Cells

**FIGURE 3:**
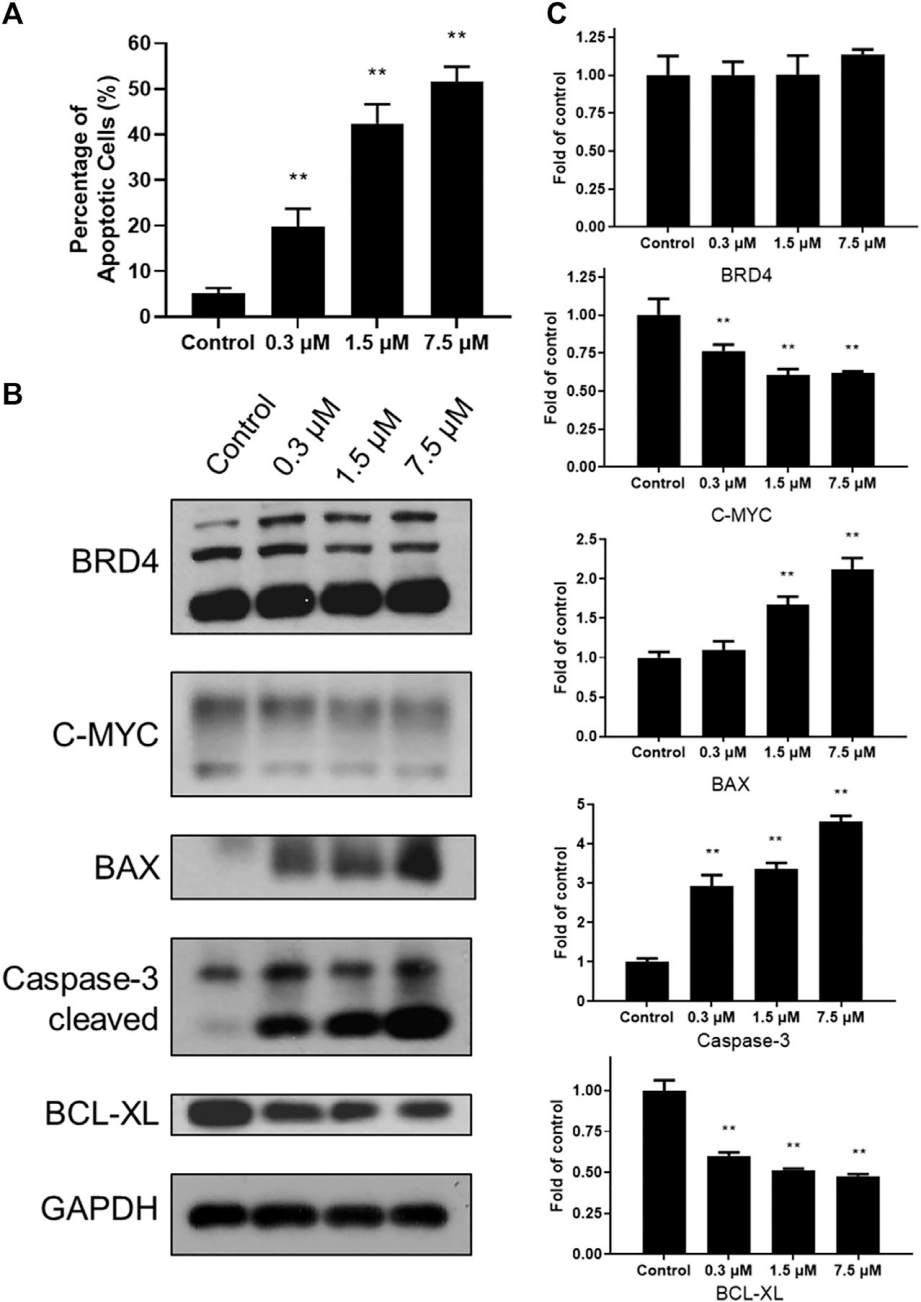
SN-38 regulated the expression of apoptotic related protein and induced apoptosis of K562 cells. **(A)** The apoptotic percentage of K562 cells treated with different doses of SN-38; **(B)**, **(C)** Expression levels of apoptosis-related proteins in K562 cells treated with different doses of SN-38 for 48 h. Data were shown as mean ± SD with three times replication. **p < 0.01 were considered statistically significant compared to control group.

To explore cytotoxicity of SN-38 in K562 cells, apoptotic analysis was also performed with Annexin V-FITC/PI double staining and quantitated by flow cytometry. SN-38 treatment of K562 cells dose dependently increased the percentage of the apoptotic population up to 19.68, 42.43, and 51.65%, respectively, compared to control (5.56%) (Figure 3A). To further explore the mechanism, expression of BRD4 was investigated when cells were exposed to SN-38 for 48 h. As shown in Figures 3B, C, SN-38 does not change the expression of BRD4. Nevertheless, substrate of BRD4 (Delmore et al., 2011), C-MYC, was decreased in a dose dependent manner. On the other hand, protein levels of cleaved caspase-3 and BAX were upregulated upon SN-38 treatment, while the apoptosis inhibitor BCL-XL was decreased. Therefore, it was confirmed that SN-38 can inhibit BRD4 and decrease the expression of its substrate C-MYC in cells, meanwhile SN-38 can regulate apoptotic related protein expression and induce cell apoptosis.

## Conclusion

In summary, with a small compound library screening, SN-38 was characterized to bind and inhibit BRD4 with IC_50_ = 660.2 nM against BRD4 (BD1), and IC_50_ = 547.7 nM against BRD4 (BD2) using drug repurposing strategy. Additional dialysis experiment and dilution assay gave a support that SN-38 can inhibit BRD4 (BD1) and BRD4 (BD2) in a reversible manner. Further cellular studies showed that SN-38 can stabilize BRD4 in cells, indicating that SN-38 can bind BRD4 in cellular level. Meanwhile, we found that SN-38 can significantly suppress the proliferation of human leukemic cell K562 with IC_50_ = 0.8138 μM for 48 h treatment, IC_50_ = 0.3285 μM for 72 h treatment, IC_50_ = 0.2798 μM for 96 h treatment, respectively, while the clinical applied BRD4 inhibitor OTX015 inhibited K562 proliferation with IC50 = 11.342 μM for 72 h treatment (Coudé et al., 2015), indicating the superiority of SN-38 as a dual targets inhibitor. Based on the molecular docking study, SN-38 was found to interact with first bromodomain of BRD4 and form strong hydrogen bond with TYR97 and PRO82, respectively, indicating the importance of carbonyl and hydroxy group of SN-38, this information may support the medicinal chemists to further optimize SN-38 as a dual target inhibitor. In addition to these findings, SN-38 was confirmed to induce the apoptosis in a dose dependent manner and inhibit the expression of BRD4 substrate C-MYC, and induce the cleavage of caspase-3 and expression of BAX. Meanwhile, it can downregulate the expression of BCL-XL. All of our findings gave an evidence that SN-38 may serve as a new potent and reversible BRD4 inhibitor in biochemical level and cellular level, and may be used for the treatment of human leukemic cell.

## Data Availability

The original contributions presented in the study are included in the article/Supplementary Material, further inquiries can be directed to the corresponding author.
