# Corrigendum: 7-Ethyl-10-Hydroxycamptothecin, A DNA Topoisomerase I Inhibitor, Performs BRD4 Inhibitory Activity and Inhibits Human Leukemic Cell Growth

**DOI:** 10.3389/fphar.2021.757060

**Published:** 2021-08-30

**Authors:** Airong Wang, Lingling Li, Mengya Li, Shujuan Wang, Chong Wang

**Affiliations:** Department of Hematology, The First Affiliated Hospital of Zhengzhou University, Zhengzhou, China

**Keywords:** SN-38, BRD4, inhibitor, leukemic cell, growth

In the original article, the title was incorrectly written as “**Case Report: 7-Ethyl-10-hydroxycamptothecin, a DNA topoisomerase I inhibitor, performs BRD4 inhibitory activity and inhibits human leukemic cell growth**”.

The correct article title is “**7-Ethyl-10-hydroxycamptothecin, a DNA topoisomerase I inhibitor, performs BRD4 inhibitory activity and inhibits human leukemic cell growth**”.

The authors state that this does not change the scientific conclusions of the article in any way. The original article has been updated.

